# Carbon Catabolite Repression in Filamentous Fungi

**DOI:** 10.3390/ijms19010048

**Published:** 2017-12-24

**Authors:** Muhammad Adnan, Wenhui Zheng, Waqar Islam, Muhammad Arif, Yakubu Saddeeq Abubakar, Zonghua Wang, Guodong Lu

**Affiliations:** 1 State Key Laboratory of Ecological Pest Control for Fujian and Taiwan Crops, Fujian Agriculture and Forestry University, Fuzhou 350002, China; 2151904006@m.fafu.edu.cn (M.A.); wenhuiz@fafu.edu.cn (W.Z.); waqarislam@m.fafu.edu.cn (W.I.); 2141904001@m.fafu.edu.cn (M.A.); ay.saddeeq@gmail.com (Y.S.A.); wangzh@fafu.edu.cn (Z.W.); 2 Key Laboratory of Bio-Pesticides and Chemical Biology, Ministry of Education, Fujian Agriculture and Forestry University, Fuzhou 350002, China

**Keywords:** carbon catabolite repression, sensing and signaling pathway, phosphorylation, hexokinase, transport proteins, cAMP, *CreA*, ubiquitination

## Abstract

Carbon Catabolite Repression (CCR) has fascinated scientists and researchers around the globe for the past few decades. This important mechanism allows preferential utilization of an energy-efficient and readily available carbon source over relatively less easily accessible carbon sources. This mechanism helps microorganisms to obtain maximum amount of glucose in order to keep pace with their metabolism. Microorganisms assimilate glucose and highly favorable sugars before switching to less-favored sources of carbon such as organic acids and alcohols. In CCR of filamentous fungi, CreA acts as a transcription factor, which is regulated to some extent by ubiquitination. CreD-HulA ubiquitination ligase complex helps in CreA ubiquitination, while CreB-CreC deubiquitination (DUB) complex removes ubiquitin from CreA, which causes its activation. CCR of fungi also involves some very crucial elements such as Hexokinases, cAMP, Protein Kinase (PKA), Ras proteins, G protein-coupled receptor (GPCR), Adenylate cyclase, RcoA and SnfA. Thorough study of molecular mechanism of CCR is important for understanding growth, conidiation, virulence and survival of filamentous fungi. This review is a comprehensive revision of the regulation of CCR in filamentous fungi as well as an updated summary of key regulators, regulation of different CCR-dependent mechanisms and its impact on various physical characteristics of filamentous fungi.

## 1. Introduction

Fungi are responsible for causing devastating diseases of fauna and flora. Pathogenic fungi have developed genetic mechanisms and molecular strategies to survive unpredictable scenarios and establish effective disease conditions in their hosts [1,2,3]. Regulation of carbon metabolism is very important for disease establishment by filamentous fungi. Carbon Catabolite Repression helps microorganisms to precisely adapt their physiology to the environment. The importance of this fine tuning is illustrated by the finding that within populations there may be balancing selections for individuals with fast and slow rates of reprogramming [2,4,5]. CCR switches off certain enzymes required to utilize less-favored carbon sources when a more readily available carbon source is present in the medium [1,5]. Therefore, CCR acts as an economical instrument in microorganisms for screening glucose. Apart from regulating the uptake of glucose, CCR may also influence the survival of microorganisms by affecting virulence, adaptation, cellular communication and motility. 

CCR has been studied extensively in *Saccharomyces cerevisiae*, which serves as a model organism for understanding the complexity of repression, derepression and induction of gene expression in response to glucose [2,6]. Among filamentous fungi, CCR has been studied most extensively in the model organism *Aspergillus nidulans*, which serves as a second model organism for comparison [7]. Mutants of CCR in *A. nidulans* have been mapped to define the genes *CreA* (Cre = catabolite responsive elements), *CreB*, *CreC* and *CreD* [8,9,10,11]. In addition, other species of *Aspergillus* such as *A. oryzae* and *A. flavus* have also been adequately studied in relation to CCR [12,13].

CreA is a transcription factor, and like Mig1 in *S. cerevisiae* it has a C_2_H_2_ Zinc finger DNA binding domain required for CCR [5]. When glucose is detected by *A. nidulans*, CreA represses the genes that encode the enzymes that utilize other secondary carbon sources such as lignocellulose [1,5,14]. It is already known that *CreA* repression occurs at transcriptional level; however, this aspect needs further work [5,15]. The regulation of cellular activities is dependent on CCR under normal circumstances, which is further regulated by *CreA* and its counterparts and also involves ubiquitination and phosphorylation. Addition and removal of ubiquitin molecules help in the activation of CreA. Moreover, CreB-CreC deubiquitination complex is also involved at this juncture [5]. Interaction of DUB enzymes with ubiquitin ligases and their combination control the quantity of transcription factors in CCR [16]. Similarly, phosphorylation controls the localization and function of CreA, by post-transcriptional modification [5]. Although CreA and MIG1 appear to be orthologs, there are obvious differences in the pathways governing CCR, as such pathways have diverged over evolutionary time. An understanding of the events in the divergence of such a complex regulatory network as CCR will provide an insight into the evolutionary mechanisms that allow rewiring of interconnected regulatory networks. In order to develop biotechnological processes, particularly for plant biomass deconstruction for conversion to high-value products, a more complete understanding of CCR in a wide variety of fungal species is needed [17,18,19].

## 2. Sensing and Signaling Pathways of Carbon Catabolite Repression

### 2.1. Yeast

The yeast, *S. cerevisiae*, has served as a model organism regarding CCR sensing and signaling cascades [20]. There are three main sensing and signaling pathways, which are interconnected and responsible for regulation of glucose metabolism in yeast [21]. The primary signal for triggering regulatory process is glucose itself [2,22]. Environmental signals are sensed by the G protein-coupled receptors (GPCRs) or other unknown proteins, leading to the regulation of downstream pathways. The glucose-signaling pathways are cAMP/PKA (consisting of Ras and the model Gpr1/Gpa2), Rgt2/Snf3-Rgt1 and the main repressor pathway Mig1-Hxk2 involving Snf1 [2,23,24,25,26].

**Figure 1:**
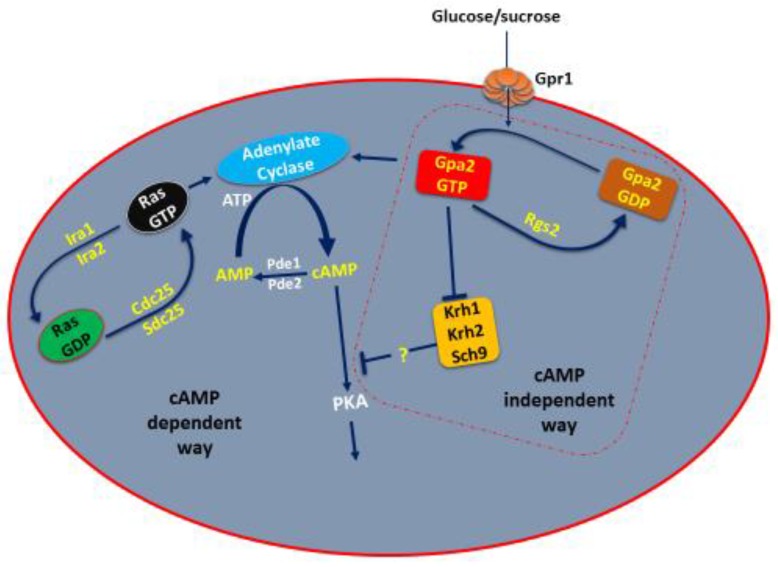
The PKA/Ras-cAMP Pathway in yeast. Adenylate cyclase is controlled by two G-Protein Systems in yeast. Ras1 and Ras2 need Sdc25 and cdc25 for activation. Ras Proteins are inactivated (Green) by Ira1 and Ira2. GPR1 system includes sucrose and glucose sensors Gpa2 and GPR1, which stimulate the adenylate activity. Krh1, Krh2, and Sch9 interact with the active Gpa2 (red), and seems to inhibit PKA activity by an unknown mechanism. Pde1 and Pde2 regulate cAMP concentration.

Activation of heterotrimeric and Ras proteins results in modulation of cAMP (cyclic adenosine monophosphate) levels upon a shift from low to high glucose. Although it is unclear how the G protein-coupled receptor (GPCR), Gpr1, senses glucose, the Gα homolog, Gpa2, interacts with adenylate cyclase. Adenylate cyclase is more strongly controlled by Ras protein, which is further regulated by cdc25 and Ira proteins (the controlling elements of adenylate cyclase) (Figure 1) [27,28]. This pathway operates under environmental conditions where PKA activation neither involves adenylate cyclase nor cAMP, but includes the Gβ-like proteins krh1, krh2 proteins and Sch9 protein kinase [29,30,31]. Therefore, in this pathway, Gpr1 and Gpa2 will activate PKA through krh1, krh2 and Sch9 proteins in a cAMP independent way (Figure 1). 

Entrance of glucose into the yeast cells is achieved by facilitated diffusion through hexose transporters (including *HXT1–HXT17*, *GAL2*, *SNF3* and *RGT2*). The uptake of glucose from the environment is efficiently coordinated through the action of transporters of differing affinities. The activity of the transcriptional repressor (*Rgt1*) is controlled by the glucose sensors Snf3 and Rgt2. Rgt2 (low-affinity glucose sensor) senses high concentrations of glucose and is induced when glucose level is high within the medium, while Snf3 is a high-affinity glucose sensor and capable of inducing high-affinity transporters when the level of glucose is very low [32]. Rgt1 is a repressor of *HXT* genes in response to Snf3 and Rgt2 signaling. Moreover, different levels of PKA will affect phosphorylation of Rgt1 and influence its binding and blocking of HXT promoters [33,34,35]. Consequently, high glucose levels will cause complete activation and increased level of PKA, which leads to phosphorylation of Rgt1, therefore low affinity HXTs (HXT1, HXT3) will be induced [36]. On the other hand, low levels of glucose lead to weak activation of PKA and in this case only high affinity HXTs (HXT2, HXT4) can be induced [37].

Phosphorylated glucose can cause repression of many genes that are involved in alternate carbon source utilization e.g., gluconeogenesis and respiration through CCR. This mechanism involves transcriptional repression in the presence of glucose and relieves repression upon limited glucose levels. The signal required for glucose repression is phosphorylated glucose and hexokinases cause this phosphorylation. Yeasts possess three hexokinases Glk2, Hxk1, Hxk2, which can phosphorylate glucose (Table 1) [21,38]. In *S. cerevisiae*, the presence of only one of these enzymes is required for growth on glucose [21]. However, only Hxk2 acts in glucose sensing and can interact with the Mig1 repressor complex [34]. 

**Figure 2:**
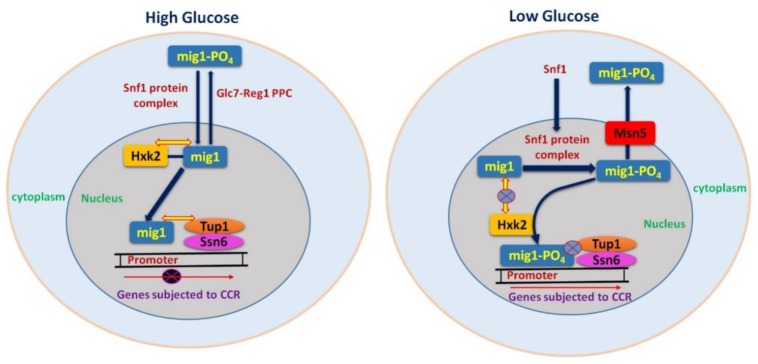
CCR regulation mechanism in *S. cerevisiae.* 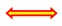 represents protein interaction in the nucleus, mig1 and hxk2, Ssn6-Tup1 co-repressor complex and 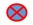 mig1. Shows that there is no protein interaction, mig1 and hxk2, Ssn6-Tup1 co-repressor complex and mig1 and between Ssn6-Tup1 co-repressor complex and promoter gene. 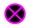 represents the inhibition of transcription [34,45,46,47,48]. During high glucose conditions, inactive Snf1 cannot cause phosphorylation of Mig1 and cellular movement of Mig1 is dependent on Glc7–Reg1 complex; whereas during low glucose levels Snf1 will be active and cause direct repression of Snf1, which will be unable to repress the CCR subjected genes.

The major components of CCR in yeast are Glc7 (protein phosphatase), Snf1 protein kinase complex and Mig1 transcriptional repressor complex as shown in Figure 2 [24,26,39]. The DNA binding protein Mig1 forms a complex with the co-repressor proteins Tup1 and Ssn6 (Figure 2) in order to carry out repression of diverse gene families and related transcriptional regulators, such as MalR (maltose utilization), Gal4 (galactose utilization), Cat8 (gluconeogenic genes), and Hap4 (respiratory genes). Glucose-regulated subcellular localization of Mig1 is very important for its proper function in repression. Mig1 quickly moves to the nucleus in repressing glucose levels, and binds to the promoters of glucose repressible genes [22,40]. However, under glucose starvation, Mig1 is mobilized back to the cytoplasm due to phosphorylation by Snf1 [40].

Snf1, is the central element of signaling for glucose repression. The Snf1 kinase complex, during glucose-limiting conditions reprograms the transcription of metabolic genes required for alternate carbon sources such as sucrose, maltose and galactose. Moreover, it impacts the remodeling and restructuring of chromatins and adaptation of cells to stress [41]. Snf1 kinase activity is activated at low glucose levels and inhibited in the presence of high glucose levels [40]. Snf1 causes phosphorylation of Mig1, and moves it to the cytoplasm during glucose starvation [34,40]. The role of Glc7 in CCR is antagonistic to that of Snf1 [39,42] (Figure 2). Glc7 plays its role in controlling various processes and has regulatory subunit Hex2/Reg1 for glucose-specific repression [42,43]. Hex2/Reg1 causes inhibition of Snf1 kinase by dephosphorylation depending upon glucose signal [26,42,44]. Therefore, the exquisite preference of glucose for yeast as a carbon source lays in its numerous, sophisticated mechanisms that it has developed to identify the presence of glucose and to adjust to multiple cellular functions accordingly.

### 2.2. CCR of Filamentous Fungi and Differences with S. cerevisiae

Glucose sensing among filamentous fungi involves GPCR and Gα subunits [57,58,59]. These proteins play similar roles in activating cAMP, irrespective of their functional differences in signaling pathways among various filamentous [60,61]. GPR4, the GPCR in *N. crassa*, plays a similar role as Gpr1 of yeast (activates adenylate cyclase in response to glucose and causes increment of cAMP) [60]. However, this protein couples with Gα1 subunit, as compared to Gα3 (a heterotrimeric protein of yeast). Gα3 subunit of *Botrytis cinerea* and *A. nidulans* plays an important role in cAMP signaling, carbon nutrient sensing and conidial germination [52,53]. Similarly, cAMP production in *Cryptococcus neoformans* is controlled by Gα3 subunit [56]. The cAMP-dependent protein kinase A (PKA) plays an important role in CCR and fungal growth by regulating primary metabolism and CCR. PKA has two catalytic subunits encoded by *PkaA* and *PkaB* in *A. nidulans*, where a major role is played by *PkaA* (via Ras proteins and GPCR) inside the cell [59,62]. Adenylate cyclase, after activation by Ras protein and GPCR pathway, leads to increased cAMP production, which binds to PkaA and releases active catalytic subunit which phosphorylate downstream targets [59,63]. The PKA activity in *A. fumigatus* increases upon the presence of glucose [64]. In *A. fumigatus*, deletion of the gene coding for *pkaC1* or *pkaC2* (catalytic subunit of the PKA), reduces fungal growth on glucose, which further supports that PKA plays a significant role in the fungal glucose metabolism [65]. 

The role of hexokinases is very prominent as only one gene encoding hexokinase is enough for growth on glucose in filamentous fungi. *GlkA* and *frA*/*hxkA* encode catalytic hexokinases in *A. nidulans* and *A. fumigatus* [11,66,67]. Unlike yeast, either one of glkA or frA can cause glucose mediated CCR repression in *A. nidulans*, and both have to be deleted to block repression [66,67]. *Rco3* of *N. crassa* is a glucose transporter homolog that has an extended C terminus [49], like Rgt2 and Snf3. Rco3 was first studied in *N. crassa* where its deletion led to a significant reduction in conidiation in submerged cultures having high levels of glucose, xylose, glycerol, or fructose [68]. The Rco3 mutants show altered response during low and high level glucose transport activity [49]. Based on these observations, it was proposed that Rco3 also functions as a glucose sensor. Mutation of MstA (high-affinity glucose transporter) also result in altered expression of other genes encoding hexose transporters in *Aspergillus niger* (mstF expression is reduced while mstC is increased) [69].

**Figure 3:**
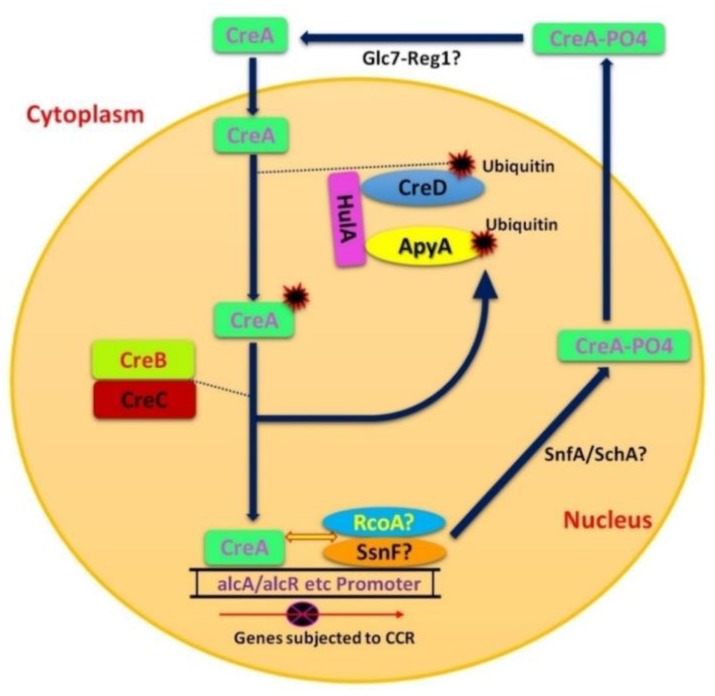
Proposed CCR regulation mechanism in *Aspergillus nidulans*. Model representing interaction of CreA, CreB, CreC, CreD, HulA, ApyA, RcoA, SsnF, SnfA and Glc7-Reg1 complex in CCR. CreD along with HulA/ApyA is required in conjugating ubiquitin and CreA. ApyA forms strong protein-protein interaction with HulA than CreB [8,57]. However CreD and ApyA are both present in *A. nidulans.* CreB-CreC complex is required to remove ubiquitin from CreA–Ub complex so that CreA can repress CCR subjected genes. CreB helps in the removal of ubiquitin from CreA to prevent degradation of CreA by proteasome [8,78,79]. The role of RcoA and SsnF in binding with the promoters of glucose repressible genes along with *CreA* is still unclear [75,80]. However the deletion of RcoA and SsnF can be lethal for *A. nidulans* [74]. SnfA and SchA can play synergistic or overlapping role in regulating CreA derepression [31].

CCR of filamentous fungi has many similarities to that of yeast, but there are some glaring differences as well. The mechanism of CCR is more complex in filamentous fungi as compared to *S. cerevisiae*, reflecting the differences in lifestyle and ability of filamentous fungi to utilize a broad range of C-sources, such as pentoses. In contrast to *S. cerevisiae*, CCR in filamentous fungi is not only regulated by glucose, but also by high concentrations of other monosaccharides that also affect nuclear localization of CreA orthologs [70]. The role of Gα subunits and GPCR are quite similar, however, the interaction of GPCR in filamentous fungi with signaling components still remains uncertain [60,61]. Relief of CCR in filamentous fungi occurs only in the presence of inducers and during metabolic stress or carbon limitation, while it is not the case in yeast [70]. *CreA*, the most important factor of CCR in *A. nidulans* is a homologue of yeast *Mig1*. As with *S. cerevisiae* Mig1, phosphorylation also affects the localization and function of CreA [71]. The presence of cellulose activates SnfA (homologue of yeast snf1), which causes CreA phosphorylation, and moves it to the cytoplasm (inactivation of CCR) (Figure 3). This is in contrast to yeast, where phosphorylation moves Mig1 into the nucleus (Figure 2). The presence of glucose inactivates SnfA, resulting in dephosphorylated CreA which returns to the nucleus [72].

In *A. nidulans* ubiquitination is involved during CCR, while in *S. cerevisiae* there are no such regulatory genes for this mechanism. Activation of CreA involves addition and subsequent removal of ubiquitin, mediated by CreD-HulA ubiquitin ligase complex and the CreB-CreC DUB complex, respectively (Figure 3) [3,73]. The co-repressor proteins, Tup1 and Ssn6, of yeast have RcoA and SsnF homologues in *A. nidulans* respectively [74]. RcoA is a WD repeat protein of *A. Nidulans* and is a homologue of Rco1of *N. crassa* [75]. CCR in *S. cerevisiae* involves the co-repressor *Tup1*, while the role in CCR of the orthologous Rco1 of *N. crassa* has not been established. Rco1 does regulate pleiotropic development and gene expression [76,77]. Similarly, in *A. nidulans*, deletion of RcoA does not eliminate CCR, but does alter chromatin structure of carbon catabolite repressible promoters (*alcA*, *alcR* and *prnD-prnB*) [74]. Interestingly, deletion of *SsnF* (the putative *RcoA* partner) is lethal in *A. nidulans* [74]. Therefore, it can be proposed that RcoA may play an indirect role in CCR by virtue of its chromatin modulation activity. Therefore, a cohesive picture of glucose signaling has yet to emerge in filamentous fungi.

## 3. Key Regulators of CCR in Filamentous Fungi

The role of the key regulators of CCR has already been briefly described above. However, we will try to provide a comprehensive description of these factors here. 

### 3.1. CreA/Cre1

In most fungal species, the zinc finger transcription factor CreA/CRE1/Mig1 mediates different aspects of CCR [5,80,81,82]. *CreA* has been identified in many filamentous fungi such as *Metarhizium anisopliae*, *Sclerotinia sclerotiorum*, *B. cinerea*, *Neurospora crassa* and *Cochliobolus carbonum* [83,84,85]. *CreA*-encoded protein contains zinc finger of Cys2-His2 class, common S (T) PXX motifs, alanine rich region and acidic domain [70,86]. It binds to 5′-SYGGRG-3′ on DNA, and the amino acid sequence of its zinc finger region is similar to yeast Mig1, and Wilms tumor suppressor protein [15].

CreA proteins of all fungi contain a highly conserved region of 42 amino acids which is rich in proline, threonine and serine residues [87]. In *T. reesei* and *Aspergillus* spp. a conserved region of *CreA* was identified to be essential for growth on carbon, lipid and nitrogen sources [5]. In *A. nidulans*, *CreA* plays a particular role in regulating the utilization of arabinan, xylan, ethanol and proline [5,16]. CreA mutants of *A. nidulans*, show changes in enzyme activity, depression of primary metabolism and metabolite profile change [88]. In filamentous fungi, *CreA* is also related to important pathways of growth and polarity. CreA of *A. nidulans* binds to the promoter region at the DNA sequence 5′-SYGGRG-3′ of xlnA and xlnD (xylanase encoding genes) and causes their direct repression [15]. The expression of xlnR, which encodes the major inducer of xylanases, and to some extent cellulase-encoding genes (Table 2) is also repressed by *CreA* [4,15,89]. All genes which are regulated by xlnR can be indirectly repressed by CreA [86]. The transcription factors, AraR and ARA1 for arabinose utilization genes (Table 2), can also be repressed by CreA [5,86,90]. 

*CreA* mediates various alternative carbon-utilizing systems, because it is a global regulator of CCR. The gene *AreA*, encodes a protein AreA that is a positive regulator and causes ammonium derepression [91]. In case of any loss of functional mutation in *AreA*, the strain will be unable to grow on d-glucose and alternative nitrogen sources since the enzymes dependent on ammonium will not be expressed during ammonium repression. However, in a few instances proline and acetamide act as nitrogen and carbon sources and their enzymes are subjected to ammonium repression and CCR [91]. In the absence of d-glucose, the *AreA* mutant strains can grow on media containing acetamide or proline as the only sources of carbon and nitrogen, respectively, but they cannot grow on d-glucose as the only source of carbon and nitrogen [91,92]. It can therefore be supposed that carbon and nitrogen repression are closely related phenomena. 

CreA regulation, both at transcriptional and post transcriptional levels, need further attention as little work has been done on this aspect [5,15,93,94]. Ubiquitination and phosphorylation cause pre- and post-transcriptional modification which may control the localization as well as function of CreA [5]. Phosphorylation of Cre1 in *T. reesei* is essential for DNA binding though Cre1 is not apparently regulated by Snf1 homolog [95,96]. For this, we propose that there are multiple binding motifs among *CreA*/*Cre1* target promoters e.g., in *A. nidulans alcR* promoter there are nine CreA binding sites [97,98], also, *H. jecorina xyr1* promoter possesses ten Cre1 binding sites [99]. Deletion of CreA binding sites from *aguA* promoter of *A. niger* enhanced the gene expression [100]. However, it was not predicable that the promoters only need the presence of 5′-SYGGRG-3′ motif or it may require additional regulatory factors necessary for binding solely to *CreA/Cre1*. In *N. crassa*, Cre1 not only binds to the promoter region motifs but also competes with the regulatory factors during cellulolytic conditions [101,102]. The strains, which have lost functional mutation of Cre1 homologs, show morphological problems while growing on carbon-rich sources. Various studies regarding the role of CreA have been confined to the transcription level regulation during CCR. However some studies suggest a more cell-wide regulatory role of this transcription factor [4,11,93]. Recent observations have revealed that CreA is not a direct target of CreB, though CreA is a phosphorylated protein but there was no evidence of its ubiquitination [73]. Despite this, the molecular mechanisms of CreA regarding its repression activity as well as CreB–CreC interaction with this regulatory setup still remain largely ambiguous [3]. 

### 3.2. CreB and CreC

Ubiquitination plays an important role during CCR by altering protein function during macromolecular assembly. Ubiquitin acts as a marker either by modifying the function of a protein or tagging it for destruction by specific proteasome [79]. Deubiquitination enzymes activate specific transcription factors by targeting their domains. The ubiquitination ligases and deubiquitination enzymes interact with each other and control the transcription factors of CCR mechanism [5,16,73]. *CreB* of *A. nidulans* is a homologue of human *UBH1* gene, which is involved in ubiquitination during CCR [78]. CreB contains 6 DUB coiled regions for recognition of the substrate, and 4 PEST sequences which act as a signal for proteolysis [78,79]. However, *CreC* is a regulatory gene which encodes a 630 amino acid polypeptide rich in proline near its N-terminus and has five WD 40 repeat motifs at its C-terminus [3,142]. Protein-protein interaction is facilitated by the WD 40 repeat regions, which form a propeller-like region [143].

*CreC* has a regulatory mechanism in multicellular eukaryotes, which is suggested by the presence of *CreB* and *CreC* homologues in mouse and humans but not in *S. cerevisiae* [81,142]. Co-immunoprecipitation experiments have shown that CreB and CreC proteins function together as a complex during repression or derepression condition. It was proved that CreB-CreC deubiquitination complex has its role in CCR as deletion of *Cre2* and *CreB* in *T. reesei* and *A. oryzae* respectively enhanced the secretion levels of hydrolytic enzymes [13,144]. The CreB-CreC deubiquitination complex eradicates ubiquitin moieties from CreA and other substrates thereby modifying the protein [79]. However, CreB overexpression partially compensates the deficiency of CreC, but overexpression of CreC does not compensate for CreB deficiency suggesting that CreB acts downstream of CreC protein. We found that *CreC* in *Magnaporthe oryzae* plays an important role in vegetative growth, conidiation and appressorium formation. *CreC* mutation hinders penetration and reduces infection, which results in attenuated virulence. *CreC* mutants represented sensitivity towards allyl alcohol in the presence of glucose and utilization of secondary carbon sources was not fully repressed by 2-deoxyglucose, which affects CCR. The genes encoding cell wall degradation enzymes such as feruloyl esterase, β-glucosidase and exoglucanase in *MoCreC* mutants were also upregulated. Therefore, it can be depicted that CreC, in addition to gene regulation, plays a much wider role in growth, development as well as virulence of the fungus [3]. 

### 3.3. CreD

CreD is involved in the ubiquitination of CreA after which CreB-CreC DUB complex comes into play (Figure 3) [8]. *HulA* is a homologue of *Rsp5* (ubiquitin ligase of yeast) in *A. nidulans* and so, CreA might be ubiquitinated by CreD-HulA ubiquitination ligase complex which may change the conformity of CreA protein in order to be targeted by proteasomes (Figure 3) [8,73]. Boase and Kelly [8] found another gene, *ApyA*, which is relatively similar to *CreD* but shows stronger interaction with HulA than CreD. Therefore, it can be supposed that *ApyA* is a result of gene duplication during evolution and it could possibly play its role in ubiquitination (Figure 3). 

CreD may act opposite to the CreB-CreC DUB complex as *CreD34* suppresses some mutant phenotypes of *CreB* and *CreC* [8]. In a diploid strain *CreD34* is recessive to *CreD*; however, genetic analysis depicts that *CreD* has a close connection with *CreC* on chromosome11 [145]. *CreD* encrypts a protein that has arrestin N and arrestin C domains, two PXY motifs and one PPXY motif when cloned by complementation and physically analyzed [8]. The PPXY and PXY motifs are respectively rich in proline and basic sequences. These features are found in transcription factors and are actively involved in protein-protein binding [146,147,148]. The mutation in *CreD34* inhibited other aspects of mutational *CreB* and *CreC* phenotypes, such that the derepression of genes *alcA* and *facA* as analyzed through the allyl alcohol and fluoroacetate sensitivity found in *CreC 27* strains [145]. The CreD34 mutant strain shows greater resistance to flouroacetamide and glucose than the wild type and this seems that *CreD34* mutant has higher repression of CCR enzymes. The suppression of the *CreB* and *CreC* mutant phenotypes by *CreD34* concludes that *CreD* has implications in reverse process to deubiquitination role of the CreB-CreC protein complex [8]. 

### 3.4. Snf1

Phosphatases and protein kinases help in the addition or removal of phosphate group to their target proteins and regulate their structure, function, and localization. They can play a major role in many processes such as functional regulation, metabolism, cell fate and secretion [71]. The protein kinase Snf1 stands for “Sucrose Non Fermenting”, which was named after a mutant unable to ferment sucrose but still can ferment glucose [40,149]. The gene *Snf1* was first studied in *S. cerevisiae*, which is a homologue of the mammalian cyclic Adenosine Mono Phosphate (cAMP)-dependent Protein Kinase AMPK [150]. It plays a significant role in sensing energy status, and its homologs are present in all eukaryotes such as fungi, plants, and animals [150,151]. *Snf1* controls CCR, by encoding a protein kinase that functions in the glucose derepression pathway in *S. cerevisiae*, while in some plant pathogenic fungi it plays vital roles in regulating and repressing the Cell Wall Degrading Enzymes (CWDE) [6,23,152].

Snf1 causes phosphorylation of its downstream repressor Mig1 and also helps in the derepression of glucose-repressed genes during low level of glucose [152]. Therefore, when glucose is depleted from the medium, *Snf1* releases the CCR. On the other hand, a high level of phosphorylated glucose inactivates snf1, making it unable to phosphorylate Mig1, and Mig1 will remain occupied in the nucleus [5,70]. Interestingly, Casein kinase II causes the phosphorylation of Cre1 transcription factor of *T. reesei* at Ser241 within its acidic domain; this is an essential post-transcriptional modification for DNA binding and repression by Cre1 [95]. However, Snf1 homolog of *Hypocrea jecorina* (*T. reesei*) was unable to phosphorylate Cre1 homologue [96]. Also, there is no solid evidence of direct phosphorylation of CreA in *A. nidulans*, but some findings propose that kinases can play an important role in controlling CreA cellular localization [5]. Even under glucose-rich conditions, deletion of two kinases, *SchA* (homologue of S. cerevisiae *Sch9*) and *SnfA* (homologue of *Snf1*), keeps CreA occupied within the nucleus [31]. 

The *Snf1* ortholog in *U. maydis* has functional similarity with *Snf1* of *S. cerevisiae*, and its mutant surprisingly produced higher expressions of pectinases and endonucleases than the wild type under derepression conditions, which means that it negatively regulates these genes. In filamentous fungi, is still unclear whether there is a conserved mechanism for carbon catabolite derepression by *Snf1*; even if it exists, the Snf1 pathway may still control some developmental processes as well as the gene expression of CWDEs [153]. 

## 4. Chromatin Modification and CCR 

Gene regulation also involves a second level of complexity ruled by epigenetics and access to chromatin (such as acetylation, methylation, and histone modification) [154,155,156]. Successful CCR operations require proper access to heterochromatin in order to control gene expression, generally organized through CCAAT box (Hap complex), acetylation levels and methylation [157,158,159,160]. The Hap complex is believed to be significant for the generation of an open chromatin structure, which enables full transcriptional activation of certain promoters [137,161]. The CCAAT-binding complex was first described in *S. cerevisiae*, and it consists of Hap2, Hap3, Hap4, and Hap5 proteins. Their homologues have been identified in different filamentous fungi such as *N. crassa* (HAP5), *T. reesei* (HAP2, 3, 5) and *A. nidulans* (AnCF) [134]. In filamentous fungi, CCAAT sequences lie in the promoters of respiratory genes, cellulase, hemicellulose and ligninolytic encoding genes [162]. 

In *Cbh2* of *T. reesei*, the presence of this sequence in the promoter region was found to be necessary for gene expression [135]. Furthermore, CRE1, HAP complex, and an unknown GTAATA binding protein affects nucleosome positioning, and influences the accessibility to the TATA box for transcription initiation of cbh2 (Table 2) [161]. During repression conditions, CreA/CRE1 directly affects chromatin structure (acetylation, methylation, nucleosome position, packaging etc.) in *T. reesei* and *A. nidulans* [163,164,165,166]. CRE1 of *T. reesei* organizes the local chromatin structure or nucleosome positioning in the xyr1 promoter as well as in the cellulases cbh1and cbh2 during repressing conditions; however its loss results in less dense chromatin structure [164,165,166]. In *A. nidulans* CreA was found to cause chromatin remodeling through histone deacetylation [163]. Gene expression is also affected by acetylation levels, which influence the access to chromatin. Consistent with this, deletion of the histone acetyltransferase, GCN5, in *T. reesei* severely affects the acetylation levels, which results in impaired growth, morphogenesis, and expression of cellulase-encoding genes [167].

## 5. Impact of CCR Components on Fungal Behavior

The components of CCR are integrated for regulation of fungal growth, development, and pathogenesis, where glucose status is one signal that conveys information about the environment.

### 5.1. Regulation of Fungal Growth

Cell-wide regulatory role of CCR is most extensively studied among *Aspergillus* spp., *T. reesei* and *N. crassa* [16,168]. *CreA* has significant influence in fungal development and growth as it reduces the activity of b-galactosidase in *A. nidulans*. Ilyés et al. [169] have observed that the growth rate of *A. nidulans* is dependent on CCR. Although in bacteria and *S. cerevisiae* growth rate and CCR are dependent on application of glucose dilutions, which has considerable effect on the growth rate, but the Crabtree effect in yeast and CCR mechanism of bacteria are different from filamentous fungi [170,171,172]. CreA mRNA represented an intricate expression profile during northern blot analysis. Monosaccharides when added to carbon deficient culture of *A. nidulans* stimulated CreA transcript formation within a short period of time. The above findings suggest that, in order to achieve CCR, a higher transcript level of *CreA* is essential and depends on glucose transportation as well as product of *CreB* gene at least partially [173]. *Snf1* plays a significant role in the growth, development and sporulation of yeast, depending on different nutrient signals [174]. Similarly, the homolog of *Snf1* in *M. oryzae* (*MoSnf1*) functionally restored the growth defects of yeast *Snf1* mutant [153].

### 5.2. Regulation/Utilization of Non-Glucose Sources

Filamentous fungi degrade plant cell wall polymers such as cellulose, hemicellulose and lignin by secreting hydrolytic enzymes [101,110,175,176,177,178,179]. The production of these enzymes is reduced or suppressed when glucose is easily available in the environment due to suppression or repression of genes encoding these enzymes [82]. Therefore, during enormous availability of glucose, the CWDE production is repressed by CCR [104]. CWDE activity is under control of glucose repression in *Cochliobolus carbonum* [85,180]. Deletion of *ccSnf1* (*Snf1* homolog) in *C. carbonum* resulted in down regulation of CWDE [180]. Interestingly, deleting *FoSnf1* in vascular wilt pathogen *Fusarium oxysporum* produced similar results [181]. In addition to regulating gene transcription, Snf1 also regulates the transport and storage of carbohydrates and fatty acids through phosphorylation of related proteins [182]. 

There are lots of reports on *CreA* regulation of CWDEs [85,153,180,183]. *CreA* homologue in *F. oxysporum* regulates the CWDEs and isocitrate lyase production [67,70,184]. In *T. reesei* and *A. nidulans*, *CreA*/*Cre1* cause gene regulation in xylan, xylose, arabinose, ethanol and proline utilization [5,16,185]. The transcriptional regulation of genes by CreA is in a “double-lock” manner [183,186]. The genes for ethanol regulation in *A. nidulans* consist of two major structural genes *aldA* (aldehyde dehydrogenase), *alcA* (alcohol dehydrogenase) and transacting regulatory gene *alcR* [97,187,188,189]. AlcR is directly repressed by CreA and alcA and aldA are repressed when *CreA* competes with alcR binding promoter sequence [98,186,190,191].

Likewise, *CreA* represses the xylanolytic system by direct repression of *xlnr* and *xlnA*, which are pathway-specific regulator and structural genes respectively [15,89,185]. *A. niger* has four genes involved in xylose regulation, which are *faeA* (feruloyl esterase gene), *xlnB* (endoxylanase gene), *aguA* (α-glucuronidase gene) and *xlnD* (β-xylosidase gene). The xylanolytic genes are generally repressed in the wild type strains while *CreA* mutants show higher xylose accumulations [15,89,185]. *T. reesei* (*H. jecorina*); *Cre1* represses the cellulose degrading enzymes by binding the cellobiohydrolase 1 *(cbh1)* promoter region, and also represses xylanase activity by binding to the *xyn1*promoter [15,99,164,185,192,193,194]. *XprG* is a transcriptional activator of extracellular protease, PrtA. However, production of extracellular protease remains unaltered following the supplementation of exogenous protein in the medium, irrespective of carbon status. Extracellular protein level increases in a carbon-deficient medium only due to mutations in *CreA*, *CreB*, *CreC* [11]. 

### 5.3. Virulence

The factors involved in CCR of filamentous fungi may directly or indirectly play their roles in pathogenicity on insects, plants and animals. In order to penetrate and enter the host plant cell, fungi release chitinases, esterases and proteinases that degrade the cells cuticle [195,196]. Virulence in entomopathogenic fungi can be increased by over expressing the chitinase Bbchit1 and subtilisin-like protease Pr1A [197]. Enzymes for cuticle degradation in *Metarhizium anisopliae* might be regulated by *CRR1* which is equivalent in function to *CreA* of *A. nidulans* [198,199]. CreA from *Beauveria bassiana* named *BbCre1* is involved in carbon source uptake, conidiation, and can impact on virulence by regulating penetration mechanism [200]. Severe symptoms may result due to overexpression of CCR encoding genes in *Alternaria citri* (black rot of citrus fruit) [1]. Wild type strain of *A. citri* only showed symptoms inside the central region of fruit, while the overexpression mutant of *A. citri* (*AcCreA*) produced severe symptoms in the central region as well as juice sac areas [1].

CreA homologue in *F. oxysporum* functions along with F-box protein (Frp1) in order to regulate carbon utilization, and most importantly required for the fungal pathogenicity [67,70,184]. *Cre1* mutation in *F. oxysporum* f. sp. *lycopersici* can restore pathogenicity defects of *Frp1* deletion mutant (because *Frp1* is required for pathogenicity of *F. oxysporum* f. sp. *Lycopersici* on tomato). The *Frp1* mutant does not express the cell wall degradation genes and ICL1 (a gene encoding an enzyme for assimilation of C2 carbon sources) [184]. This suggests that both Frp1 and Cre1 proteins are significant for regulating transcription of CCR genes. In *M. oryzae*, there are three mediators of CCR that are inhibitor proteins, namely Nmr1-3, sugar sensor Tps1 and multidrug and toxin extrusion (MATE)—family pump mdt1. The activities of MATE-Family, *Nmr1-3*, and *Tps1* are crucial for glucose metabolism during the infection process. Glucose-6-phosphate is sensed by Tps1, which inactivates Nmr1-3 and initiates the CCR. Glucose assimilation is regulated by *Mdt1*, which is also important for pathogenicity, sporulation and nutrient utilization. The mutational studies of all the three mediators showed early expression of CWDEs, which suggests their critical role during pathogenicity of *M. oryzae* [201,202]. 

Deletion of nearly 21 genes except *CreA*, *XlnR*, and *Snf1* kinase homologs did not affect the virulence in *Alternaria brassicicola* (necrotrophic fungal pathogen). *A. brassicicola* causes the black spot disease of Brassicas. *A. brassicicola* expresses CWDEs in a unique way as deletion of only *XlnR* but not *CreA*, *Snf1* or *Ste12* can reduce the ability of glucose utilization [203]. *Verticilium dahliae* (soil borne fungi causing vascular wilt) *VdSnf1* is a sucrose non-fermenting 1 gene that regulates CCR. The *VdSnf1* mutant was unable to produce CWDEs, and had considerably reduced growth on galactose or pectin as compared to xylose, sucrose or glucose medium. Moreover, *VdSnf1* is considered important for CCR and colonization of cotyledons, stem and roots of tomato and eggplant [204]. The pathogenicity of *C. carbonum* was also reduced on deletion of *ccSnf1* (*Snf1* homolog) [180]. The snf1 deletion mutant of *M. oryzae* (Dmosnf1) showed reduced pathogenicity, few and abnormally shaped conidia than the wild type which suggest that it plays vital role in sporulation and pathogenicity of *M. oryzae* [153].

In almost all analyzed fungi, deletion of *CreA* can result in impaired colony morphology, with few aerial hyphae and spores [102,205,206]. While CreA mutation in the case of *P. chrysogenum* and *F. oxysporum* can be lethal for these fungi [3,207]. We have also observed that *M. oryzae*, *CreA* null mutants grow slower than the wild type in a glucose-independent manner, which suggests some faults in CCR. The CCR defects were further supported by resistance of CreA mutants to 2-deoxyglucose and inefficient glucose transport. In addition, production of conidia was reduced, conidial germination and appressorium formation were delayed which resulted in less virulence among the mutants (non-published data). Recently, we found that *MoCreC* plays significant role in growth, conidiation and pathogenicity of *M. oryzae* [3]. Currently, Beattie et al. [208] proposed a model for the role of *CreA* and disease progression of invasive aspergillosis, which describes that CCR in an environmental filamentous fungus is dispensable for the initiation of pulmonary infection but essential for infection maintenance and disease progression. They revealed that fungal fitness and invasion of microenvironment requires the support of CCR mediated genetic network in *A. fumigatus*.

## 6. Concluding Remarks

CCR has optimized carbon utilization as an evolved trait in filamentous fungi [209]. CCR provides clues on how filamentous fungi cope with different nutrient situations. The genome machinery of CCR may involve various factors such as *CreA*, *CreB*, *CreC*, *CreD*, *HulA*, *ApyA*, *RcoA*, *SsnF*, *SchA* and Glc7-Reg1 [8,40,73]. CreA activity is regulated via ubiquitination and deubiquitination [5]. Although the roles of CreB, CreC and CreD are well established in relation to CreA activation, some ambiguities still exist regarding the functions of *HulA* and *ApyA* in ubiquitination of CreA [73]. Formerly, it was accepted that CreB-CreC deubiquitination complex causes activation of CreA necessary for its repression [79]. However, recent studies revealed that CreA is not a direct target of CreB; instead, CreA is a differentially phosphorylated protein with no evidence of ubiquitination [73]. It will be of immense significance to further investigate the link between CCR subjected genes and CCR complex (CreA, RcoA, SsnF) among the filamentous fungi. The role of SchA/SnfA in phosphorylating CreA and its movement towards the cytoplasm is not very convincing. Another very important aspect that needs more attention in CCR of filamentous fungi is the role of Glc7-Reg1 in dephosphorylation and mobilization of CreA back to the nucleus. Gene regulation ruled by epigenetics adds another level of complexity controlled by factors such as acetylation, methylation, and histone modification for successful operation of CCR [155,156]. In the future, dissecting the operation of CCR at the transcriptome, proteome and metabolome levels will be of fundamental importance to produce valuable insights for understanding CCR of filamentous fungi. 

## Figures and Tables

**Table 1:** Glucose sensing comparison between *S. cerevisiae* and filamentous fungi.

Nature/Kind of Glucose Sensor	Sensor/Sensors in *S. cerevisiae*	Sensor/Sensors in Filamentous Fungi	Function in Filamentous Fungi
Transporter	Snf3, Rgt2	Rco3 (*N. crassa*)	Rco3 in *N. crassa* might perform the same role as compared to yeast proteins but structure of Rco3 is different. It may have wider role than yeast proteins in CCR which only regulate hexose transporters [49].
Hexokinase	Glk2, Hxk1, Hxk2	HxkA, GlkA (*A. nidulans*)	No evidence showed direct role of a single hexokinase like yeast to start CCR but phosphorylation is required [50].
		HxkC, HxkD (*A. nidulans*)	Show response in carbon starved conditions while not involved in CCR [51].
G-protein coupled receptor	Gpr1, Gpa2 (Gα3)	Gpr4, GCNA1-3 (Gα1-3) (*N. crassa*)	In response to glucose the G protein coupled receptor GPR4 play role in cAMP signaling. While GPR4 in yeast interacts with Gα1 instead of Gα3, and Gα1 through Gα3 play role in nutrient sensing.
		BCG3 (Gα3) (*Botrytis cinerea*)	During germinating conidia has function in cAMP signaling/carbon sensing [52].
		GanB (Gα3) (*A. nidulans*)	During germinating conidia has function in cAMP signaling/carbon sensing [53].
		GasC (Gα3) (*Penicillium marneffei*)	Has no role in nutrient sensing but essential for germinating conidia [54].
		Gpr4 (*C. neoformans*)	Homologous to yeast Gpr1 but has no role glucose signaling [55].
		Gpa1 (Gα3) (*Cryptococcus neoformans*)	Involved in cAMP signaling in response to glucose and melanin production [56].

**Table 2:** Transcription factors of filamentous fungi responsible for carbon source utilization.

Transcription Factor	Major Role	Fungi
CreA/CRE1	CCR	*Aspergillus* spp., *N. crassa*, *Trichoderma* spp., etc. [70]
BglR/COL-26	Sugar sensing and regulation of glucosidase expression	*N. crassa*, *Trichoderma reesei* [103,104,105].
VIB1	C-derepression, may also play role in cellulases induction	*N. crassa* [104]
CLR-1/ClrA	Cellulose utilization	*N. crassa*, *Aspergillus* spp. [106,107,108].
CLR-2/ClrB/ManR	Cellulose utilization	*N. crassa*, *Aspergillus* spp., *Talaromyces cellulolyticus*, *Peniciulium oxalicum* [106,107,108]
ACE2, ACE3	Cellulose utilization	*T. reesei* [109,110]
McmA	Cellulase regulation	*A. nidulans* [111]
ACE1	Cellulase repression	*T. reesei* [112,113]
XlnR/XLR1/XYR1	Hemi-cellulose utilization	*Aspergillus* spp., *Fusarium* spp., *Trichoderma* spp., *M. oryzae*, *Protubera* canescens, *N. crassa*, *T. cellulolyticus* [89]
WC-1/BLR1, WC-2/BLR2	Hemi-cellulose utilization	*Trichoderma* spp., *N. crassa* [114,115,116]
HCR-1	Hemi-cellulase repressor	*N. crassa* [117]
AraR	l-Arabinose utilization	*Aspergillus* spp. [118,119]
ARA1	l-Arabinose utilization	*M. oryzae* [120]
AmyR	Starch utilization	*Aspergillus* spp. [121,122]
MalR	Maltose utilization	*Aspergillus* spp. [123,124]
ClbR	Cellobiose utilization	*Aspergillus aculeatus* [125,126]
GalR	d-Galactose utilization	*A. nidulans* [127,128]
GalX	d-Galactose utilization	*Aspergillus* spp. [129]
GaaR	Galacturonic acid utilization	*B. cinerea*, *Aspergillus niger* [130,131].
GaaX	Galacturonic acid repressor	*A. niger* [131]
XPP1	Xylanases repressor	*T. reesei* [132,133]
HAP complex	Carbohydrate-Active enZymes regulation (CAZy regulation)	*Aspergillus* spp., *Trichoderma* spp., *N. crassa* [134,135,136,137]
RhaR	l-Rhamnose utilization	*Aspergillus* spp. [138,139]
InuR	Inulin utilization	*A. niger* [140,141]
